# Exploring Anabolic Androgenic Steroid Use Among Cisgender Gay, Bisexual, and Queer Men

**DOI:** 10.1001/jamanetworkopen.2024.11088

**Published:** 2024-05-14

**Authors:** Eric Kutscher, Arslaan Arshed, Richard E. Greene, Mat Kladney

**Affiliations:** 1 Division of General Internal Medicine, Icahn School of Medicine at Mount Sinai, New York, New York; 2 NYU Grossman School of Medicine, New York, New York; 3 Bellevue Hospital Center, New York, New York

## Abstract

**Importance:**

Anabolic androgenic steroids (AAS) are disproportionately used by sexual minority men, with the physical and mental health implications of AAS use incompletely understood.

**Objective:**

To understand the reasons for use and health care needs of gay, bisexual, and queer cisgender men using AAS.

**Design, Setting, and Participants:**

This qualitative study was conducted from November 2021 to May 2023 using self-administered questionnaires and semistructured interviews that were transcribed and coded using reflexive thematic analysis. Participants were recruited through convenience and snowball sampling from lesbian, gay, bisexual, transgender, and queer clinical centers in New York, New York, as well as through online platforms. All patients self-identified as cisgender and gay, bisexual, or queer.

**Exposures:**

History of nonprescribed AAS use for a minimum of 8 consecutive weeks was required.

**Main Outcomes and Measures:**

The primary outcomes were reasons for and health implications of AAS use and interactions with health care practitioners, as determined through interviews. Interview transcripts were collected and analyzed.

**Results:**

Thematic saturation was reached after interviews with 12 male participants (mean [SD] age, 44 [11] years), with the majority of participants identifying as gay (10 participants [83%]), White non-Hispanic (9 participants [75%]), being in their 30s and 40s (9 participants [75%]), holding a bachelor’s degree or higher (11 participants [92%]), and having used steroids for a mean (SD) of 7.5 (7.1) years. One participant (8%) self-identified as Black, and 2 (17%) identified as Hispanic. Seven men (58%) met the criteria for muscle dysmorphia on screening. Nine overarching themes were found, including internal and external motivators for initial use, continued use because of effectiveness or fear of losses, intensive personal research, physical and emotional harms experienced from use, using community-based harm reduction techniques, frustration with interactions with the medical community focused on AAS cessation, and concerns around the illegality of AAS.

**Conclusions and Relevance:**

In this qualitative study, AAS use among cisgender gay, bisexual, and queer men was found to be associated with multifactorial motivators, including a likely AAS use disorder and muscle dysmorphia. Despite all participants experiencing harms from use, men seeking medical help found insufficient support with practitioners insistent on AAS cessation and, thus, developed their own harm reduction techniques. Further research is needed to assess the utility of practitioner education efforts, the safety and efficacy of community-developed harm reduction methods, and the impact of AAS decriminalization on health care outcomes for this patient population.

## Key Points

**Question:**

How and why do cisgender gay, bisexual, and queer men use anabolic androgenic steroids (AAS), and what are their unique health care needs?

**Findings:**

In this qualitative study of 12 men, AAS use was secondary to multifactorial motivators, including a likely AAS use disorder and muscle dysmorphia. Despite all participants experiencing harms from use, men seeking medical assistance found practitioners to be insistent on AAS cessation and, thus, developed their own harm reduction techniques.

**Meaning:**

Cisgender gay, bisexual, and queer men using AAS reported insufficient medical support, suggesting that further research is warranted on the utility of practitioner education, the safety and efficacy of community-developed harm reduction methods, and the impact of AAS decriminalization on health care outcomes for this patient population.

## Teaser

This qualitative study examines nonprescribed use of anabolic androgenic steroids among gay, bisexual, and queer cisgender men in New York, New York.

## Introduction

Anabolic androgenic steroids (AAS) are synthetic testosterones used by approximately 2.9 to 4.0 million US residents at least once, with 1 million developing AAS dependence.^1^ AAS use disproportionately impacts gay, bisexual, and queer (GBQ) men, with 5.0% to 13.5% of gay and bisexual men reporting use, and up to 25.0% considering use.^2,3^ Although many motivations exist for AAS use, improving physical appearance and gaining muscle and strength are the most common.^4^ Among GBQ men, the history of HIV and prescriptions for AAS for HIV wasting syndrome,^5^ views on masculinity,^6^ gender role strain and internalized homophobia,^7^ increased pressures to obtain an ideal male physique, and overemphasis on appearance and desirability^8,9^ are cited as additional factors.

Despite widespread use, the health impacts of AAS remain incompletely understood. Although AAS is associated with increased risk of acquiring hepatitis B and C viruses,^10^ its role in coronary artery disease,^11,12^ stroke,^13,14^ and the risk of developing prostate cancer remain poorly characterized.^15^ Remarkably, gay men using AAS have a prevalence of HIV 39 times greater than that of the general population, although many acquire the virus before initiating AAS.^10^ GBQ men using AAS have higher self-reported rates of male-male condomless anal sex in the past year, as well as increased use of ecstasy and methamphetamines.^16^

The mental health implications of AAS are also unclear. Although a diagnosis of AAS use disorder was excluded in the *Diagnostic and Statistical Manual of Mental Disorders* (Fifth Edition) (*DSM-5*), multiple studies have found an addiction model, including loss of control, social impairment, risky use, and pharmacologic dependence, in up to 24% of individuals using AAS.^17,18,19,20,21,22,23^ AAS use has also been closely associated with muscle dysmorphia (a subtype of body dysmorphic disorder), including among cisgender sexual minority men.^24,25^

Despite these concerns, most individuals using AAS have declined to disclose their use to a medical practitioner, and general practitioners themselves often feel underprepared when caring for patients using AAS.^26^ The majority of individuals who disclose AAS use have felt discriminated against after disclosure.^4^ Treatment for AAS dependence is controversial, with accepted practice ranging from psychotherapy to harm reduction to prescribed testosterone, with the unique treatment needs of GBQ men unknown.^27,28,29^ Although facilitators to care for individuals using AAS have been investigated, the particular needs of GBQ men remain poorly defined.^30^ This project seeks to understand the lived experiences of GBQ cisgender men using AAS in the US, with a particular focus on motivations and patterns of use, knowledge-seeking behaviors, perceived benefits and harms of use, interactions with health care practitioners, and community perspectives on how practitioners can best care for patients using AAS.

## Methods

### Setting and Sample

This qualitative study used semistructured interviews and self-administered questionnaires administered between November 2021 through May 2023. Inclusion criteria included cisgender adult men who self-identified as GBQ and had used AAS for at least 8 consecutive weeks at any point of their life. Exclusion criteria included active psychotic or manic symptoms, exclusive use of prescription AAS by a practitioner with use only as prescribed, and past or current prostate cancer. Participants were recruited through convenience and snowball sampling from lesbian, gay, bisexual, transgender, and queer clinical centers in New York, New York, using posters and social media platforms. Recruitment occurred until theoretical saturation was reached.^31^

This study was approved by the NYU Langone Health and Bellevue Hospital Center Independent Review Boards. Participants gave written consent after reading a participant information form and were given a chance to ask questions before participation. In this article, identifying attributes are avoided. Data reporting followed Consolidated Criteria for Reporting Qualitative Research (COREQ) reporting guidelines.^32^

### Data Collection

Participants self-administered a demographics questionnaire and a screening questionnaire testing for muscle dysmorphia, the Muscle Dysmorphic Disorder Inventory,^33,34^ using REDCap.^35,36^ One of 2 authors with no prior patient relationship (E.K., a cisgender gay White man, or A.A., a cisgender gay South Asian man) conducted interviews either in person or through a Health Insurance Portability and Accountability Act–secured videoconferencing platform. Interviews were approximately 1 hour long, with 1 participant requesting a brief follow-up interview, and were audio recorded and transcribed using a computer-based transcription system (Otter.ai; Otter). Authors wrote field notes after each interview. All participants were compensated with $100 gift card. Participants’ race and ethnicity were self-identified and were included in this study to assess for the generalizability of our findings.

### Interview Guide Development

The authors developed an interview guide using concept elicitation questioning, highlighting topics found to be important in prior research. Two preliminary interviews were conducted with this guide, after which modifications were made to enhance the flow and clarity of interviews.

### Statistical Analysis

Interviews were evaluated through the reflexive thematic analysis approach, as described by Braun et al,^37^ following inductive reasoning. Two reviewing authors (E.K. and M.K.) familiarized themselves with the transcripts and developed coding for all interviews independently using Atlas.ti version 22 (ATLAS.ti Scientific Software Development GmbH) to categorize responses, then harmonizing their categorization to create consensus themes. The 2 reviewing authors then developed and reviewed these themes, followed by refining, defining, and naming themes. Both authors acknowledged their identity as cisgender White gay men, critically evaluating their analyses to assess for bias. Finally, themes were woven together with data extracts and shared among the entire research team for consensus. Questionnaires were reviewed, with a Muscle Dysmorphic Disorder Inventory score of 39 points considered meeting the criteria for muscle dysmorphia.^33,38,39^ Participants were not provided with interview transcripts or analysis before publication.

## Results

Twelve male participants (mean [SD] age, 44 [11] years) completed semistructured interviews until thematic saturation was reached, with the majority identifying as gay (10 participants [83%]) and White non-Hispanic (9 participants [75%]), being in their 30s and 40s (9 participants [75%]), and holding a bachelor’s degree or higher (11 participants [92%]). Of our sample, 1 participant (8%) self-identified as Black, and 2 (17%) identified as Hispanic. Participants had used steroids for a mean (SD) of 7.5 (7.1) years. During recruitment, an additional 2 participants declined to partake in the study after screening, 3 participants completed screening but were then lost to follow-up, 6 did not follow up after initially reaching out to study team, and 7 did not meet inclusion criteria; possible differences in demographics between cohort participants and those lost to follow-up are unknown. Table 1 outlines the demographics of participants. Seven of the 12 participants (58%) met the criteria for muscle dysmorphia (Table 2). Nine overarching themes with additional subthemes were found (Table 3).

**Table 1.** Participant Demographics

Characteristic	Participants, No. (%) (N = 12)
Age, y	
Mean (SD)	44 (11)
20-39	1 (8)
30-39	5 (42)
40-49	4 (33)
50-59	0
60-69	2 (17)
≥70	0
Sexual orientation	
Gay	10 (83)
Bisexual	2 (17)
Race	
Asian	0
Black or African American	1 (8)
White	11 (92)
Ethnicity	
Hispanic	2 (17)
Non-Hispanic	10 (83)
Highest level of education completed	
High school	0
Associates	1 (8)
Bachelors	10 (83)
Masters	1 (8)
Age at first AAS use, y	
Mean (SD)	35 (8)
20-39	3 (25)
30-39	5 (42)
40-49	3 (25)
50-59	1 (8)
60-69	0
≥70	0
Total duration of AAS use, y	
Mean (SD)	7.5 (7.1)
0-1	1 (8)
1-4	3 (25)
5-9	4 (33)
10-14	2 (17)
15-19	0
≥20	1 (8)
Unknown	1 (8)
Use of nontestosterone performance-enhancing drugs	
Yes	9 (75)
No	3 (25)
Use of medications to treat adverse effects from testosterone use	
Yes	10 (83)
No	2 (17)

Abbreviation: AAS, anabolic androgenic steroids.

**Table 2.** Muscle Dysmorphic Disorder Index Results

Factor	Cohort score, mean (SD)	Score for gay men nationwide, mean (SD)^a^
Desire for size	18.4 (4.1)	9.9 (4.6)
Appearance intolerance	9.9 (3.0)	11.5 (4.3)
Functional impairment	12.3 (4.9)	6.1 (3.0)
Total^b^	40.7 (9.6)	27.4 (7.7)

^a^ Scores are reported from Nagata et al.^34^

^b^ Scores greater than 39 are considered positive for muscle dysmorphia.

**Table 3.** Themes and Subthemes With Representative Quotations

Themes and subthemes	Quotations
Internal motivations for initiation	
Childhood weight problems	“I had a weight problem my whole life. Starting when I was a young kid…kind of being pretty heavy, pretty overweight…. So being able to, like be shirtless places is important because…image wise, it’s to not be the fat kid anymore.”
	“I was the skinny sneezy theater nerd…. So, I feel like this is kind of like the revenge against that.”
Body dysmorphia	“I think it’s mostly it’s body dysmorphia that’s driving this abuse.”
Unrealistic body ideals	“I know that I tried to shape myself into an ideal that I have in my head. I know that parts that are not realistic and parts that are unfair. And I know that parts of that are not attainable.”
Cravings for youth	“I just want to be out and about. So from the from research I did it seemed like having increased testosterone could help with my healing and my aches and pains and give me more energy.”
Insecurity from sexual orientation	“I think that a lot of gay men have body dysmorphia, and they don’t like the way they look…they feel not good enough because of their sexual orientation. So, I think when you combined that with, you know, feelings of inadequacy, whether they were not accepted by their family, or their parents, or whatever the case is, there’s always that subconscious, like, they’re not good enough. And so, they feel like if they if they tried to make themselves genetically as best as possible, they could somehow overcome that inadequacy.”
External motivations for initiation	
Introduction through partners	“I was dating someone who was using it for his workouts. And he was he was happy with his results.… And throughout the course of dating him eventually I started using it as well. Didn’t know really anything about it only what he told me and the doses that he gave me....”
Peer pressure	“I feel like it’s everybody does it? I mean, most of a lot of our peer group is muscle queens, so it’s very normalized.”
Upcoming events	“We were going to go on a gay cruise, and my husband at the time really wanted to do a cycle of steroids because we were in the circuit group of friends, and some of them have large muscle bodies.”
Work related	“I started working in adult film, and expectation in adult film is that you’re supposed to look like basically like Superman. Like you have to be like in shape…. And so, I wanted to be constantly ready for that…. And part of that means taking the anabolic steroids.”
Iatrogenic	“My first boyfriend had been part of a medical study at [redacted] Hospital [redacted], that was studying the effects of testosterone on aggression and aggressive behavior…. A physician there named [redacted] suggested that I might try it. And I had never used steroids at all before.”
Positive reinforcement from steroid use	
Satisfaction with results	“I just think back to how I was like, five years ago…I’m happier with myself, I feel way, way more confident in just pretty much every element of my life.”
	“The great thing about steroids is that they work.”
Treating muscle dysmorphia	“Even your inner demons or, or self-hatred, like kind of melts away whenever you’re getting constant verbal and physical affirmation from all these other insanely hot men…which is what I wanted to get out of it, and is absolutely something that happened.”
Dissatisfaction with results	“My steroid use started out pretty okay, and then it became at some point, like a crutch, like addictive. And I totally went into like body dysmorphia mode and wanted to do more and more and get bigger and bigger. And it is like, almost like an addiction.”
Enhanced energy or elation from use	“I want to do more testosterone because the feeling of doing it is really incredible. Not to the end of like a drug or something...[but] it makes me feel really good while I’m on it.”
Increased libido	“A blast is basically going through puberty 3.0. You’re way hornier…which actually worked out well being a gay male.”
External validation	“People are complimentary of the way I’m looking. So that’s obviously a big ego boost and confidence booster and yeah, I feel a little bit more free to do some of the things that I want to do.”
Moving up the social ladder	“People talk to me, it’s easier to make friends, people will come up to me in public for jobs, leadership roles. People give me money…. I can do things that other people wouldn’t be allowed to do just because of my size and the way I look.... When I first pinned [injected], we make a joke about the echelons of hotness and how certain levels of hardness will grant you certain privileges and how I’m starting to slowly reach the upper echelon and how I can do things that other people wouldn’t be allowed to do just because of my size and the way I look.”
	“I do feel like gay men are very much visually oriented, physically oriented, there’s a lot of competition…to be the cream that rises to the top, you know…the best, the strongest, the most successful…having either the money and the resources or the body or the position, the title for some people.”
Fear of discontinuation of steroid use	
Fear of losing gains	“For me, they’ve almost become a necessary evil. Even if I do like low amounts, I almost feel like I can never get the results that I used to have without steroids.”
Fear of confronting unenhanced self	“If somebody has shaved their head for a decade, and they know that their hair is thinning, they’re afraid to grow their hair out, because they’re afraid, they would see just how bald they really are. So they should continue shaving. And I get a feeling that that’s what I’m sort of experiencing is like, I’ve been doing this for so long now that I’m afraid of what would happen.”
Fear of withdrawal	“When you stop, you have to experience for about a month, you’re going to lose energy, you’re going to have no libido, you’re going to be sad. And so like, I knew that was coming.”
	“If you stopped putting steroids in your body, and just stopped, It’s like putting not putting oil or gas in a car—your body crashes.”
Intensive research	
Online	“There’s a lot of wikis and you know, I look at the Reddit forums a lot for wiki information. And you try to find relatively non skeezy looking sites get information about steroids and stuff like that.”
Peers	“I’ll ask my dealer friend who is very, very knowledgeable. And he’s a personal trainer. So and he uses them himself. And he’s tried everything and he sells, so he knows his firsthand experience.”
Medical research	“I’ve searched journals for you know, adult research on testosterone…I legit do search medical journals.”
	“From what I’ve generally like read and seen, telmisartan has a large, like, positive effects for heart health. I was like it, I think, kind of extends to like, some other areas, but like with evidence particular to telmisartan it like, there’s been, like, there’s been shown some like good, good effects for actually reversing left ventricular hypertrophy. Which, like, is known to be a very, like a very non trivial health risk steroid use…if I can be proactive there, I want to be…the risks of a small dose of ARB are not significant, considering they’re like the most prescribed medicines in the country.”
Health care adjacent support	“We have a doctor friend who kind of does the same thing who kind of explained to us what we need to do, and gave us some things we needed to us, and told us exactly what we needed to get from Amazon for it to be successful.”
Physical and emotional harms from AAS	
Musculoskeletal	“I had an instant feeling of being stronger. And this is a very upsetting thing that happened. I injured myself in the gym. This is two days after I took my very first dose. And it was so stupid. Like I think I was just I had I felt a big mental rush and a feeling of being much stronger. And I actually ruptured a bicep…. I ended up having to have surgery. And then of course, I couldn’t even go to the gym for several months.”
Cardiovascular	“I was terrified, because I’ve never been put under and had my heart shocked before…. Steroid use is why I’m here with my chest partially shaved. And sitting in a chair, terrified, because I was going to be knocked out and then my heart was going to be shocked. And I was responsible.”
Infectious	“One day, I didn’t put a Band-Aid on. And I got an infection in my butt and I had to like call my doctor friend and get him to prescribe me Doxy because I was very worried.”
Aesthetic	”You have a shimmer because of all the body oil, but it tore up our skin...acne, okay. And my husband’s a brown guy. So he had a lot of scarring as well. So that was pretty damaging.”
Mental and emotional health (ie, “roid rage”)	“I would probably say it cost me one or two relationships…. It’s been depression, borderline sex addiction. I don’t know all the things that come along with those types of emotional or mental illnesses.”
Worsening muscle dysmorphia	“People tell me I look huge, and I don’t see it in the mirror. Or I’ll tell my husband he looks amazing. He’s like, ‘I don’t see it.’ It’s like literally, it’s in your brain. It’s the kind of thing you cannot perceive how big you actually are.”
Guilt around use	“If someone asks you if you’re on steroids, you respond ‘Steroids are illegal.’ And that kind of shuts people up because it’s a non-answer…as long as you stick to that line, you’re not exactly lying, but you’re not revealing what you’re doing.”
	“You feel guilty for entertaining the vanity and…wanting to align yourself to this way of looking, way of being.”
Harm reduction techniques used	
Ensuring purity	“It’s the label…. Does it look like it was printed on a printer? Or is it are the numbers actually embossed that sort of thing to look for? Spelling mistakes, obviously, which are, I find pretty common and things that are sort of homemade.”
Injection practice	“Only order sterilized, hypodermic, single use needles from healthcare websites.”
	“I really focused on the hygiene of the area while I’m doing it and after I’m doing it and make sure always use alcohol, always use a Band-Aid, and inject in a bathroom that’s been wiped down with bleach.”
Dosing modifications (ie, cycling)	“Gently you build up and then you taper off. And then in then you use support drugs at the post cycle in order to prevent any testicular shrinkage.”
Laboratory testing	“And I like the regular blood work is a harm reduction technique also to make sure that my health isn’t spiraling out of control.”
Blood pressure monitoring	“I checked my blood pressure about once a week.”
Exercise	“I do cardio to keep my cholesterol levels [from going] through the roof.”
Avoiding other substances	“I don’t do recreational drugs, I sleep more. I don’t drink a lot of alcohol. I don’t smoke. I stay active.”
Gym safety	“I need to be smart at the gym, and I need to, you know, not stress out my tendons.”
Blood donation	“I donate blood if I donate blood every six months. If I’m on things like Equipoise [boldenone undecenoate] I kind of up to three months just because my hematocrit starts to get high on that and yeah.”
Aromatase inhibitors	“I keep an AI on hand, just in case I ever feel those negative side effects.”
External oversight	“One of the reasons I have a coach is because I know I have that that body dysmorphia, that if I don’t have someone else checking me, I’ll either overtraining or go to the gym too often, I’ll under consume or over consume food, I won’t balance it properly myself.”
Interactions with medical systems	
Stigma	“Nobody, not no one tells their doctors everything. Everyone that I’ve experienced in the gay world that’s using steroids—they don’t tell their doctors.”
	“If you get a really good if you have a really good doctor, they’re there. They have an open mind. They understand that, that what you’re doing is more of a of an art than a science. And you’ll usually get cautioned, but they’ll usually also listen It’s when they don’t listen that you’d have the most concerning.”
Lack of knowledge	“Most doctors don’t really know very much about, like, contemporary steroid usage.”
Abstinence focus	“I would say maybe 5% of healthcare providers will work with the patients, instead of just telling them to instantly stop using.”
Online clinics	“It’s becoming easier now with all the testosterone clinics, you just go online and pick one.”
	“Through the online clinic…I just pay every month and everything is all inclusive, you know, the bloodwork and everything…. They also offer…Cialis from them, like that controversial diabetes drug that people get for weight loss, you know, they could sell you that for a lot of money. So, they tend to have a lot of things.”
Importance of gay doctors	“Knowing that he’s gay, and he works in a gay community…I also knew that he would probably be safe to talk to about it.”
	“I think there tend to be gay doctors that are sort of in the know, and everybody will go to them…they’ll be okay with it. They’re very non-judgmental…probably, maybe they’re doing them too.”
Illegality of AAS	
Therapeutic vs supratherapeutic dose	“I feel like it is very much like plastic surgery. It just allows you to change your body in a very specific way.”
Fear of documentation consequences	“I don’t talk to them about use, just because insurance and it will put a nick on my file and mark me for insurance, alright, have the high risk of being a homosexual behavior. So I don’t want to add more to having my assurance kinda revoked or whatnot.”
Fear of legal consequences	“The issue here is that the fact is that these substances are illegal. And is that going to get me in trouble? Once I was absolutely sure that it would not be disclosed beyond the confines of that room or in my medical record [would I disclose].”

Abbreviations: AAS, anabolic androgenic steroids; AI, aromatase inhibitor.

### Internal Motivations for Initiation

Participants reported internal and external motivations for AAS initiation. As internal motivators, some men identified childhood obesity or thinness as reasons to pursue a more muscular body in adulthood. For others, underlying body dysmorphia predated and/or motivated AAS use. Some men noted the importance of unrealistic body ideals for GBQ men and a desire to try and achieve them. Insecurities around sexual orientation and an attempt to compensate for perceived deficits in masculinity were also reported.

### External Motivations for Initiation

As external motivators, partners and peers were frequently cited as instigators for AAS use. The pressure to obtain an ideal body was most frequently reported around preparing for event in which men anticipated being shirtless around other GBQ men (such as circuit parties and gay cruises or vacation destinations). Participants repeatedly described a community that normalized and sometimes explicitly encouraged AAS use. Multiple men highlighted their work in the adult entertainment industry as practically requiring them to use AAS to be successful.

### Positive Reinforcement From AAS Use

After starting AAS, most participants noticed gains and changes in their physique along with increased energy and libido that positively reinforced continued use. These gains were described by both participants who felt that AAS were working as intended and those who felt that higher doses or more intense AAS regimens were needed to obtain their goals.

Some participants felt that AAS use improved self-image sufficiently to combat preexisting muscle dysmorphia and body dissatisfaction, whereas others felt that AAS use made the dysmorphia worse by placing the power of easily changing body shape within their control. Most participants found increased external validation to be an important motivator for continued use and felt that AAS use allowed them to climb the social ladder within the GBQ community.

### Fear of Discontinuation of AAS

Multiple men expressed a need to continue AAS out of fear of discontinuation. Some felt ready to stop AAS but were scared to lose muscle gains, whereas others continued using to avoid confronting what their body looked like when not enhanced with AAS. When considering cessation of AAS, many reported a fear of a crash or withdrawal when stopping and thus continued use, whereas others noticed an unpleasant and unmanageable period of decreased energy from secondary hypogonadism that ultimately motivated them to reinitiate use.

### Intensive Research

Access to evidence-based and reliable information was almost universally desired, with most men conducting substantial amounts of research on AAS. However, data sources were often informal with information sourced from nonmedical websites, social media postings, and blogs. Peers, coaches, and suppliers were often relied on for guidance on effective regimens and methods to mitigate adverse effects. Notably, multiple men searched medical journals and peer reviewed research, extrapolating from these materials information that felt relevant to their own lives and AAS practices. These interpretations were often insightful, with participants able to explain biological mechanisms of action. Participant research was occasionally made possible from working as a health care practitioner, or more frequently from being close to a health care practitioner who used AAS and would provide friends with guidance and support. Overall, men desired more streamlined and reputable methods for information gathering.

### Physical and Emotional Harms From AAS

Every participant reported at least one instance of harm they attributed to AAS use. Most commonly noted harms were cardiovascular, including hyperlipidemia, hypertension, hypertrophic cardiomyopathy, and atrial fibrillation. One man reported needing a cardioversion, which he attributed to AAS use, whereas another reported developing symptoms concerning for heart failure. Two men reported tendon ruptures requiring surgery, with an additional 3 developing tendinitis requiring physical therapy. Two participants developed cellulitis at an injection site. Multiple men reported balding, acne, testicular shrinkage, and night sweats as adverse effects.

Aside from physical harms, emotional harms were reported frequently, including difficulty with anger management, emotional instability, and worsening muscle dysmorphia. One participant reported that steroids were the root cause of his recent divorce. Men reported a concern about AAS use being perceived as cheating to obtain results and insisted that hard work at the gym was the primary reason for muscle gains. Participants frequently kept their AAS use secret out of fear of judgment or embarrassment. A sense of moral distress and self-judgment was notable, with men wondering whether their use showed an overvaluing of aesthetics and superficiality.

### Harm Reduction Techniques Used

Men universally attempted to avoid or minimize AAS-related harms through harm reduction methods. Participants prioritized obtaining AAS from seemingly reliable sources, inspecting labels for any evidence of tampering or low-quality manufacturing. Men learned sterile injection techniques, obtaining supplies for injecting from health care supply warehouses, online, or through needle exchange programs. Men regularly planned schedules with cycling, or having discrete periods of steroid use followed by abstinence. At the end of a cycle, men reported using postcycle therapy (often clomiphene) to induce endogenous testosterone production and minimize the risk of secondary hypogonadism. All men routinely checked bloodwork, monitoring blood counts, kidney and liver function, prostate-specific antigen levels, and cholesterol levels. A few reported daily checks of their blood pressure or blood glucose levels. Cardiovascular exercise was often considered protective. A minority of participants hired nonmedical coaches to monitor their cycles to optimize overall safety. Most participants noted that steroid use had increased their libido and the number of sexual encounters. However, participants used preexposure prophylaxis (PrEP) for HIV prevention and attributed an increase in condomless sexual activity and increased number of sexual partners more to PrEP than to AAS use.

Participants commonly used additional nonprescribed medications to mitigate the effects of steroid use. For example, some men reported taking aromatase inhibitors and selective estrogen receptor modulators to decrease gynecomastia. Aspirin was thought to decrease the risk of blood clots and strokes from polycythemia, with participants also opting to donate blood to decrease blood counts. Many took prescribed or nonprescribed statins to decrease cholesterol. One participant purchased nonprescribed telmisartan to decrease his risk of developing hypertrophic cardiomyopathy.

### Interactions With Medical Systems

Study participants reported discontentment and distrust of the medical system, with real and perceived stigma preventing multiple participants from disclosing steroid use. For participants who did disclose, there was a sense of dissatisfaction with practitioner knowledge about the risks and benefits of steroid use and a discomfort with clinicians focusing exclusively on abstinence from AAS. For the participant who required a cardioversion, he withheld information on AAS use until moments before the procedure because of fear of judgment.

Facilitators to participant disclosure to health care practitioners were reported. First, men overall felt most comfortable talking about steroid use with doctors who themselves identified as GBQ or who had a large GBQ patient panel. Men perceived these clinicians as less judgmental and less likely to focus on AAS cessation or abstinence only. Participants also embraced online clinics where testosterone could be prescribed by a practitioner in a direct-to-consumer model.

### Illegality of AAS

When discussing how to optimize the care for individuals using AAS, the legal status of AAS as a Schedule III drug was perceived as one of the biggest barriers to care. Men were concerned that the illegality of their substance use required them to purchase from unregulated markets and to hide their use from their doctors. Participants often felt that there was an arbitrary distinction between use of AAS at physiologic vs supraphysiologic doses and that AAS use itself was more aligned with plastic surgery than with many other recreational drugs. The illegality of AAS resulted in a fear of documentation of use, with men concerned that disclosure could result in denial of disability and life insurance, refusal to prescribe AAS for secondary hypogonadism in the future, and employment consequences.

## Discussion

Overall, the themes from this qualitative study were similar to those found in previous research among cisgender men regardless of sexual orientation.^30^ Individuals start and continue AAS for many reasons, not always for underlying body and/or muscle dysmorphia. The importance of masculinity discussed by participants overlaps with machismo views on masculinity and self-confidence, as described elsewhere.^40,41,42^

Our work contributes to the literature describing the existence of an AAS use disorder, because many participants continued use despite harm owing to the efficacy of AAS and the fear of losing muscle mass if abstinent.^21,23,43,44^ This bias toward an addictive nature may be the result of our inclusion criteria requiring a minimum of 8 weeks of use yet supports the inclusion of AAS use disorder within future iterations of the *DSM*.

Our study participants did not associate their AAS use with any increase in risk of acquiring HIV. Unlike Ip et al,^10,16^ men in our study reported an increase in number of sexual partners from increased libido with steroid use, but reported consistent use of PrEP predating their AAS use, with no change in the likelihood of unprotected sex from AAS use.

Individuals using AAS desired accurate, scientifically reviewed, and comprehensive information on AAS use, with high-quality and evidence-based resources thought to be highly beneficial. The interim need to rely on peers for information instead of medical practitioners was similar to findings from other studies.^45,46,47^

Participants perceived that disclosure to health care practitioners could be more harmful to their care than therapeutic given the lack of knowledge of most health care practitioners and the repercussions of medical record documentation, thus highlighting the urgent need for enhanced practitioner education and use of empathetic approaches to care for sexual minority men using AAS.^9^ Abstinence-focused models of care for individuals using AAS did not appear to be efficacious for participants in our study, because all men experienced harm from AAS but continued to use nonetheless. Participants who disclosed AAS use often perceived messages focused on cessation as bias and discrimination, leading to decisions to forgo future medical care. Further research can assess the acceptability of more nuanced discussions about abstinence focused on the specific AAS most associated with harm, such as trenbolone.^48^

In the absence of medical harm reduction guidance, participants described community-developed harm reduction techniques, with some techniques similar to those proposed as treatments for AAS dependence.^49,50^ Community-led medical interventions have a historical role within the GBQ community, as demonstrated through the inventions of PrEP and doxycycline postexposure prophylaxis. Yet, many harm reduction techniques in our study are newly reported and require further research on their safety and efficacy.

Finally, illegality around AAS use complicates the ability for patients to obtain medical care, as similarly demonstrated by Piatkowski et al.^51^ To optimize health care outcomes, future work should revisit the risks and benefits of the scheduling of AAS, as well as test methods to prevent AAS use disclosure from the electronic medical record to life and disability insurance companies.

### Limitations

This study has limitations that should be mentioned. We had great difficulty recruiting for our study. Although 30 individuals contacted our research team with interest in enrollment, 18 participants ultimately were not included in the study owing to ineligibility, loss to follow-up, or declining to participate after initial screening. The final cohort of participants was predominantly White, college-educated gay men, despite outreach and recruitment in areas where traditionally marginalized individuals seek care. This is consistent with prior research showing that men in the US using AAS were typically White, highly educated, and with an above-average income.^52^ It is unknown whether this cohort reflects the demographics of the GBQ population using AAS given the substantial cost and knowledge that participants relied on to use AAS, or whether our results are subject to a sampling bias.

## Conclusions

In this qualitative study, AAS use among GBQ men was found to be the result of multifactorial motivators, including a likely AAS use disorder and muscle dysmorphia. Despite all participants experiencing harms from use, men seeking help found insufficient health care support from practitioners insistent on AAS cessation and, thus, developed their own harm reduction techniques. Further research is warranted to assess for the utility of physician education efforts, the efficacy and safety of community-developed harm reduction methods, and the impact of AAS decriminalization on health care outcomes for this patient population.

## Notes

Supplement.
Data Sharing Statement
