# Supplementary material for: Exploring Anabolic Androgenic Steroid Use Among Cisgender Gay, Bisexual, and Queer Men
Source: JAMA Netw Open. 2024 May 14;7(5):e2411088. doi: 10.1001/jamanetworkopen.2024.11088 (PMC11094559; doi:10.1001/jamanetworkopen.2024.11088)
Supplement: Supplement. — Data Sharing Statement [file jamanetwopen-e2411088-s001.pdf]

Data Sharing Statement

Kutscher. Exploring Anabolic Androgenic Steroid Use Among Cisgender Gay, Bisexual, and Queer Men. *JAMA Netw Open*. Published May 14, 2024.
doi:10.1001/jamanetworkopen.2024.11088

Data
Data available: No

Additional Information
Explanation for why data not available: We explicitly stated in our consent forms that data would only be used for the purposes of our study.
